# Roentgen Rays as a Remedy for Articular Rheumatism

**Published:** 1898-06

**Authors:** 


### ABSTRACTS FROM FOREIGN JOURNALS.

### GERMAN.

Under the Direction of J. Preston Hoskins,
PRINCETON, N. J.

Roentgen Rays as a Remedy for Articular Rheumatism.—The Russian medical journal *Wratsch* contains a communication by Sokolow in regard to the reported successful treatment of articular rheumatism in children. The patient, wrapped in linen cloths, was placed at a distance of from 50–60 cm. from the source of the rays and exposed to their influence from ten to twenty minutes. The first experiment was made on a nine year-old girl whose wrists, ankles, and knee-caps were severely swollen, and who suffered great pain; all these symptoms of disease totally disappeared after she had been treated twice. In the second case, that of a fourteen-year-old girl, all rheumatic pains vanished after one application of the rays. In the third case, a five-year-old girl, whose wrists and knee had been severely attacked, was cured after the affected parts had been subjected to the rays three times. In this case it was possible to observe the swelling diminish beneath the rays; this is said to have been confirmed by actual measurements made from time to time; in four days the circumference of the wrist diminished 3 cm. The last patient was a girl, thirteen years of age, that suffered from chronic rheumatism and had had heart-trouble for five years. The disease had grown worse, and the pain was very excruciating. The swelling at the knee had contracted the leg at an angle of 45°. Each time the Roentgen rays were applied to the part affected she was able to stretch the leg somewhat further, and the pain diminished; all signs of disease vanished after four applications of the rays.—*Thierärzt. Centralblatt*, February 1, 1898.

